# Effects of *Cirsium palustre* Extracts and Their Main Flavonoids on Colon Motility—An Ex Vivo Study

**DOI:** 10.3390/ijms242417283

**Published:** 2023-12-09

**Authors:** Dominika Szadkowska, Magdalena Chłopecka, Jakub W. Strawa, Katarzyna Jakimiuk, Daniel Augustynowicz, Michał Tomczyk, Marta Mendel

**Affiliations:** 1 Institute of Veterinary Medicine, Warsaw University of Life Sciences, ul. Ciszewskiego 8, 02-786 Warsaw, Poland; dominika_szadkowska1@sggw.edu.pl (D.S.); magdalena_chlopecka@sggw.edu.pl (M.C.); 2 Department of Pharmacognosy, Faculty of Pharmacy with the Division of Laboratory Medicine, Medical University of Białystok, ul. Mickiewicza 2a, 15-230 Białystok, Poland; jakub.strawa@umb.edu.pl (J.W.S.); katarzyna.jakimiuk@umb.edu.pl (K.J.); daniel.augustynowicz@sd.umb.edu.pl (D.A.); michal.tomczyk@umb.edu.pl (M.T.)

**Keywords:** *Cirsium palustre*, flavonoids, colon motility

## Abstract

For centuries, various species from the genus *Cirsium* have been utilized in traditional medicine worldwide. A number of ethnopharmacological reports have pointed out that *Cirsium* plants can be applied to diminish digestive problems. Among them, *Cirsium palustre* (L.) Scop. (Asteraceae) stands out as a promising herbal drug candidate because its constituents exhibit antimicrobial and antioxidant potential, as evidenced by ethnopharmacological reports. As a result, the species is particularly intriguing as an adjunctive therapy for functional gastrointestinal and motility disorders. Our research goal was to verify how the extracts, fractions, and main flavonoids of *C. palustre* affect colon contractility under ex vivo conditions. An alternative model with porcine-isolated colon specimens was used to identify the effects of *C. palustre* preparations and their primary flavonoids. LC-ESI-MS was utilized to evaluate the impacts of methanol (CP1), methanolic 50% (CP2), and aqueous (CP3) extracts as well as diethyl ether (CP4), ethyl acetate (CP5), and *n*-butanol (CP6) fractions. Additionally, the impacts of four flavonoids, apigenin (API), luteolin (LUT), apigenin 7-*O*-glucuronide (A7GLC), and chrysoeriol (CHRY), on spontaneous and acetylcholine-induced motility were assessed under isometric conditions. The results showed that *C. palustre* extracts, fractions, and their flavonoids exhibit potent motility-regulating effects on colonic smooth muscle. The motility-regulating effect was observed on spontaneous and acetylcholine-induced contractility. All extracts and fractions exhibited an enhancement of the spontaneous contractility of colonic smooth muscle. For acetylcholine-induced activity, CP1, CP2, and CP4 caused a spasmolytic effect, and CP5 and CP6 had a spasmodic effect. LUT and CHRY showed a spasmolytic effect in the case of spontaneous and acetylcholine-induced activity. In contrast, API and A7GLC showed a contractile effect in the case of spontaneous and pharmacologically induced activity. Considering the results obtained from the study, *C. palustre* could potentially provide benefits in the treatment of functional gastrointestinal disorders characterized by hypomotility and hypermotility.

## 1. Introduction

Disorders involving gut–brain interaction (DGBIs), formerly called functional gastrointestinal disorders (FGIDs), are a modern global threat to human health and are the most common diagnoses in gastroenterology [1,2]. Studies have shown that approximately one-third of patients referred to gastroenterology clinics have been diagnosed with DGBIs [3]. Among them, the most common are irritable bowel syndrome (IBS) and functional dyspepsia (FD). Currently, DGBIs are diagnosed and classified using criteria standardised by the Rome Foundation. The pathophysiology of DGBIs is complex and has not been completely studied. According to the biopsychosocial model created by Engel and adapted by Drossman, pathophysiology is related to a combination of motility disturbance, visceral hypersensitivity, altered mucosal and immune function, altered gut microbiota, and altered central nervous system processing [1,4]. The following are some of the mechanisms involved: the dysregulation of the immune system, inflammation, and compromised barrier function. There is now a broad consensus that individuals with a genetic predisposition are at risk of developing inflammatory bowel disease (IBD) because of a compromised intestinal epithelial barrier, which exhibits heightened permeability in tight junctions. In such instances, these individuals experience an amplified immune response in the gut, directed towards the gut microbiota. This immune response is not easily regulated, ultimately resulting in the initiation of chronic intestinal inflammation [5]. In the course of the inflammatory response, various mediators are released by immune cells, including cytokines, chemokines, and eicosanoids [6]. Research has shown that the intestinal microbiota and metabolites that they generate are significant in regulating colonic motility, secretion, and absorption; therefore, they play a role in modulating critical pathophysiological pathways in DGBIs [2,4,7]. A recent study has also revealed that microorganisms can influence visceral hypersensitivity and pain reception [8]. In addition to the gut microbiota, mast cells also contribute to the development of visceral hypersensitivity. Mast cells can induce alterations in gastrointestinal tract function, and an elevated mast cell count impacts the permeability of the intestinal mucosal barrier, thereby contributing to the emergence of visceral hypersensitivity [9]. Additionally, mast cells exhibit various functions, such as regulating secretion and peristalsis, making them a potential target for pharmacological intervention in the treatment of IBS [10].

Disorders involving gut–brain interactions cause bothersome symptoms that can significantly reduce the quality of a patient’s life, both physically and mentally [11]. Due to the complex aetiology of the problem, the available treatment methods do not completely cure the disease and thus are not entirely satisfactory, and new treatment alternatives are intensively sought [12]. With the increased popularity of herbal medicines and dietary supplements in modern society, plant extracts or their phytoconstituents offer a promising option for symptomatic therapies in patients suffering from disorders of gut–brain interactions.

*Cirsium palustre* (L.) Scop., also known as Marsh plume thistle or European marsh thistle, is an herbaceous plant belonging to the Asteraceae family. The species is native to Europe and western and eastern Siberia and was also introduced to the northern United States and Canada [13,14]. Numerous studies have been carried out on the antioxidant and antimicrobial activities of *C. palustre* [15,16,17,18,19]. The dominant antioxidants determined in *C. palustre* leaves were the following flavonoids: eriodictyol 7-*O*-glucoside, luteolin 7-*O*-glucoside, and 6-hydroxyluteolin 7-*O*-glucoside, as well as chlorogenic acid [20]. In another study, Nazaruk et al. investigated the antiproliferative effects of *C. palustre* essential oil obtained from underground parts against breast adenocarcinoma cells (MCF-7 and MDA-MBA-231). It was revealed that the essential oil exhibits moderate antiproliferative activity against adenocarcinoma. Moreover, these concentrations were below the level capable of inhibiting the proliferation of healthy cells, such as normal skin fibroblasts [21].

Traditionally, *Cirsium* plants were used to cure gastrointestinal ailments, especially diarrhoea and dysentery [22,23]. Within the Slavic ethnic groups, plants belonging to the genus *Cirsium* were employed for the management of colic or other gastrointestinal problems [24,25]. *C. palustre* is an interesting candidate for new phytomedicine due to its multiple biological activities identified thus far; however, more data are needed to clarify its possible uses in various health conditions. Although very few studies have been conducted thus far, some *C. palustre* extracts and fractions affect the motility of the swine colon [26,27], making them very promising candidates for the symptomatic treatment of disorders of gut–brain interactions. Therefore, the goal of the study was to find out if *C. palustre* flavonoids and selected preparations affected intestinal contractility patterns ex vivo.

## 2. Results

### 2.1. Phytochemical Screening of Selected C. palustre Extracts/Fractions

**Figure 1:**
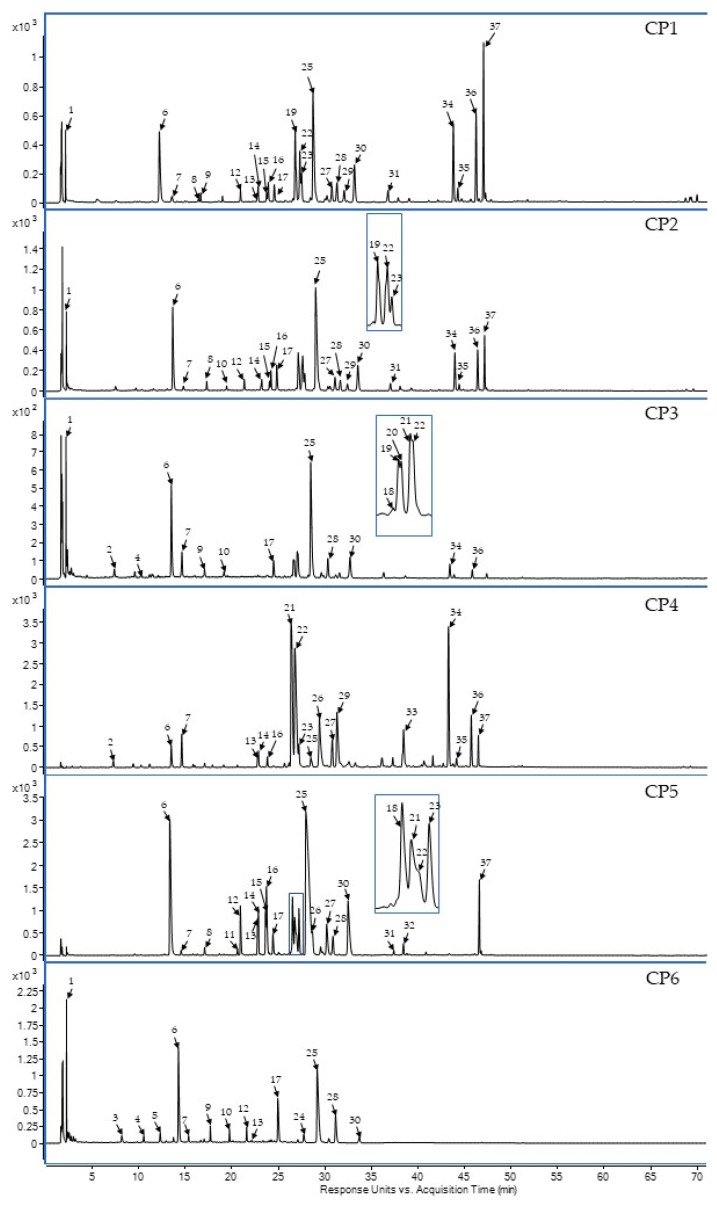
The UV chromatogram with a designation of the main metabolites of the analysed CP1-CP6 extracts, recorded at wavelength of 280 nm.

To characterise the composition of secondary metabolites, crude extracts and fractions of *C. palustre* flower heads were analysed by LC-PDA-HRMS. Thirty-seven compounds were found, including quinic acid derivatives esterified with caffeic, ferulic, and *p*-coumaric acids as well as flavones that are specific to the Asteraceae family (Table 1). The diether fraction (CP4) is largely dominated by dicaffeoylquinic acid derivatives (19–22) and aglycones, such as luteolin (33, LUT), apigenin (34, API), kaempferol (35), and chrysoeriol (36, CHRY). Apigenin 7-*O*-glucuronide (25, A7GLC) was the main component of the acetate fraction (CP5). In the *n*-butanol fraction (CP6), as in the crude methanolic (CP1) and hydro-methanolic (CP2) extracts, the predominant compounds were chlorogenic acid (6) and apigenin 7-*O*-glucuronide (25, A7GLC). Furthermore, rare isokaempferide derivatives, such as isokaempferide 7-*O*-glucoside (27) and isokaempferide 7-*O*-glucuronide (28), were found using standard substances from a previous study [28]. The results of phytochemical screening by LC-PDA-HRMS are presented in Figure 1.

### 2.2. Effect of Flavonoids and C. palustre Extracts and Fractions on the Spontaneous Contractility of Swine Colonic Smooth Muscle

**Figure 2:**
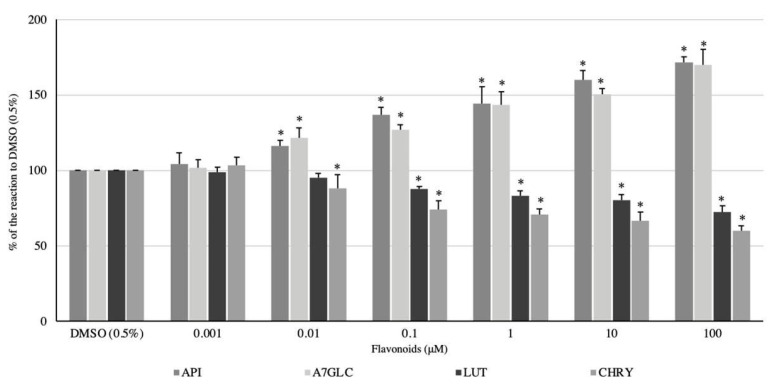
The effect of apigenin (API), apigenin 7-*O*-glucuronide (A7GLC), luteolin (LUT), and chrysoeriol (CHRY) on the spontaneous activity of swine isolated colonic longitudinal smooth muscle. The results are expressed as % of the response to DMSO (0.5%). The results are expressed as mean of 5 independent experiments (±SD); *p* ≤ 0.05 vs. DMSO (0.5%); * *p* ≤ 0.05 vs. DMSO (0.5%).

All flavonoids used in the experiment exhibited a dose-dependent effect on the spontaneous contractility of the longitudinal smooth muscle of the swine colon. While LUT and CHRY showed a spasmolytic effect, API and A7GLC enhanced the magnitude of the spontaneous motor activity. When comparing the myocontractile potency of API and A7GLC, that of the former was slightly higher. For LUT and CHRY, the latter showed a noticeably stronger spasmolytic effect (Figure 2). API clearly enhanced the magnitude of spontaneous motor activity in a dose-dependent manner. The lowest dose that induced a significant myocontractile effect was 0.01 μM, and the reaction amounted to 116.27 ± 3.63% of the control reaction. The highest magnitude of contractile response was noted when apigenin was administered at a dose of 100 μM (171.43 ± 4.0% of the reaction to DMSO). A7GLC used in a concentration range of 0.01 to 100 μM caused a clear dose-dependent enhancement of spontaneous muscle activity (Figure 2). The administration of a dose of 0.01 μM resulted in an even stronger contraction response than that observed with API at the same dose, and the response rate of the control reaction was up to 121.52 ± 6.74%. The highest magnitude of contractile response was noted when a dose of 100 μM was used and reached 171.43 ± 4.0% of the reaction produced by DMSO (0.5%). LUT exhibited a significant spasmolytic effect on the longitudinal smooth muscle of the colon in a dose range of 0.1–100 μM. The effect was dose-dependent and increased with higher substrate concentrations. The response ranged from 87.81 ± 1.65 to 72.44 ± 4.4% of the reaction to DMSO (0.5%) for LUT applied at concentrations of 0.1 and 100 µM, respectively (Figure 2). CHRY, similar to LUT, exerted a relaxing effect on colonic longitudinal smooth muscle, but in this case, the effect was significantly stronger. Additionally, the effect was dose-dependent, and the minimum dose to achieve a significant change in spontaneous contractility was 0.01 μM. The reaction ranged from 88.08 ± 9.15% to 59.91 ± 3.31% of the smooth muscle reaction to DMSO (0.5%) when CHRY was applied at concentrations of 0.1 and 100 µM, respectively (Figure 2).

**Figure 3:**
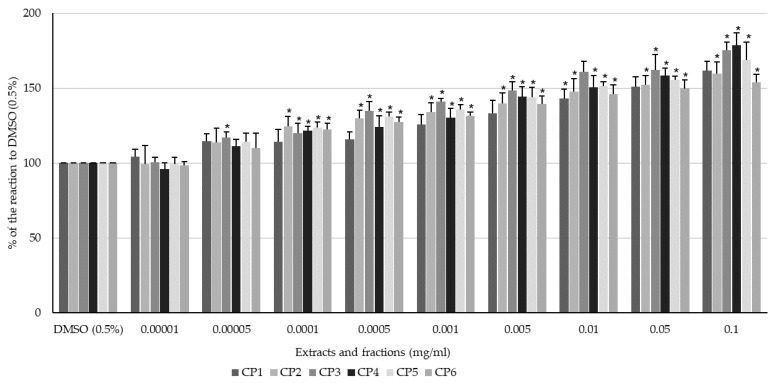
The effect of methanolic extract (CP1), 50% methanolic extract (CP2), aqueous extract (CP3), ether residue (CP4), acetate residue (CP5), and *n*-butanol residue (CP6) of *C. palustre* on the spontaneous activity of swine isolated colonic longitudinal smooth muscle. The results are expressed as % of the response to DMSO (0.5%). The results are expressed as mean of 5 independent experiments (±SD); *p* ≤ 0.05 vs. DMSO (0.5%); * *p* ≤ 0.05 vs. DMSO (0.5%).

All extracts and fractions enhanced the spontaneous contractility of the longitudinal smooth muscle of the swine colon in a dose-dependent manner (Figure 3). Among the extracts, the aqueous extract (CP3) exhibited the strongest myocontractile effect (Figure 3). For the fractions, it is impossible to clearly indicate the one that exhibited the strongest myocontractile effect. The magnitude of the reaction differed between fractions and concentrations (Figure 3). CP1 exhibited a significant myocontractile effect on the longitudinal smooth muscle of the colon in a dose range of 0.00005–0.1 mg/mL. The response ranged from 114.42 ± 5.08 to 161.62 ± 6.21% of the reaction to DMSO (0.5%) for 0.00005 and 0.1 mg/mL CP1, respectively. CP2 also caused a clear dose-dependent enhancement of spontaneous muscle activity, although the effective dose range was slightly narrower, starting at a dose of 0.0001 mg/mL. The highest magnitude of contractile response was noted when a dose of 0.1 mg/mL was used and reached 159.67 ± 7.93% of the control reaction. Utilising CP3 caused the strongest contraction of the smooth muscle of the colon among all the extracts. The reaction ranged from 116.95 (5.38%) to 175.39 (5.38%) of the smooth muscle reaction to DMSO (0.5%) when the extract was applied at concentrations of 0.00005 and 0.1 mg/mL, respectively (Figure 3). CP4 produced a dose-dependent myocontractile effect on the colon specimens if administered in a concentration range of 0.0001–0.1 mg/mL. The reaction ranged from 121.68 (2.77%) to 178.37 (8.29%) of the control reaction. CP5 also enhanced the magnitude of the spontaneous motor activity, and the minimum dose to achieve a significant change in spontaneous contractility was 0.00005 mg/mL. The reaction ranged from 113.97 (6.09%) to 168.69 (11.90%) of the smooth muscle reaction to the vehicle at 0.00005 and 0.1 mg/mL, respectively. For CP6, the lowest dose inducing a significant myocontractile effect was 0.0001 mg/mL, and the reaction amounted to 122.57 ± 4.14% of the control reaction. The highest magnitude of contractile response was noted when CP6 was administered at a dose of 0.1 mg/mL (153.58 ± 5.62% of the reaction to DMSO, 0.5%).

### 2.3. Effect of Flavonoids and C. palustre Extracts/Fractions on ACh-Provoked Contractility of Swine Colonic Smooth Muscle

**Figure 4:**
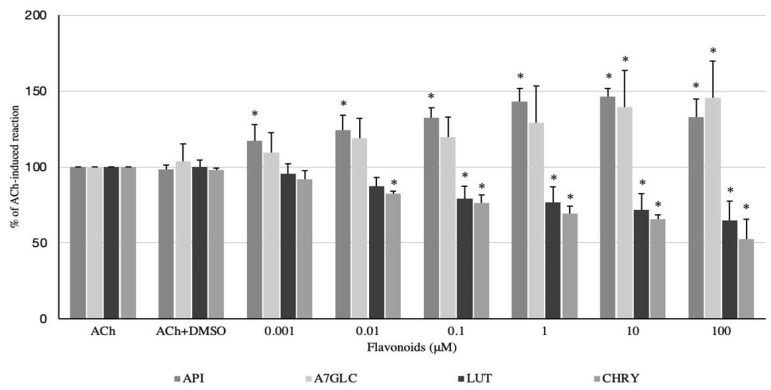
The effect of apigenin (API), apigenin 7-*O*-glucuronide (A7GLC), luteolin (LUT), and chrysoeriol (CHRY) on the ACh-induced activity of swine isolated colonic longitudinal smooth muscle. The results are expressed as % of ACh-induced contraction. The results are expressed as mean of 5 independent experiments (±SD); *p* ≤ 0.05 vs. DMSO (0.5%); * *p* ≤ 0.05 vs. ACh (10 μM).

The spasmolytic effects of luteolin (LUT) and chrysoeriol (CHRY) on ACh-induced contractions were undoubtedly more profound than the effects on the spontaneous motor activity of the colonic preparations. In contrast, apigenin and apigenin glucuronide exerted a spasmodic effect, and moreover, the range of effective doses of glucuronide was much narrower (Figure 4). The administration of API caused a myocontractile effect in a dose-dependent manner in a range of 0.001–10 μM. The response ranged from 117.32 ± 10.85 to 146.44 ± 5.24% of the reaction to ACh for API administered at concentrations of 0.001 and 10 μM, respectively (Figure 4). API at a dose of 100 μM also increased muscle contraction, but the effect was weaker (like the 0.1 μM dose) and amounted to 132.62 ± 12.12% of the reaction produced by ACh. Interestingly, A7GLC markedly increased the acetylcholine-induced response only at doses of 10 and 100 μM. The response reached 139.48 ± 24% and 145.50 ± 24.34%, respectively (Figure 4). LUT clearly weakened ACh-produced contractions of the colonic smooth muscle. As with spontaneous contractility, the myorelaxant effect occurred in a dose range of 0.1–100 μM and increased as the concentrations of LUT increased. Moreover, the effect was stronger than in the case of spontaneous motor activity and ranged from 78.92 ± 8.39% (LUT 0.1 μM) to 64.73 ± 12.78% (LUT 100 μM) of the control ACh-induced reaction. CHRY produced a dose-dependent spasmolytic effect on the colonic specimens exposed to acetylcholine in a dose range of 0.001–100 μM. The reaction ranged from 82.51 ± 1.43% to 52.53 ± 12.92% of the smooth muscle reaction to ACh for CHRY used at concentrations of 0.001 and 100 μM, respectively (Figure 4).

**Figure 5:**
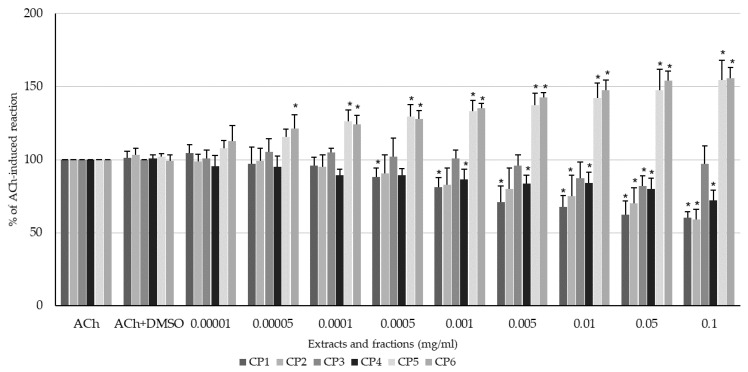
The effect of methanolic extract (CP1), 50% methanolic extract (CP2), aqueous extract (CP3), ether residue (CP4), acetate residue (CP5), and *n*-butanol residue (CP6) of *C. palustre* on the ACh-induced activity of swine isolated colonic longitudinal smooth muscle. The results are expressed as % of ACh-induced contraction. The results are expressed as mean of 5 independent experiments (±SD); *p* ≤ 0.05 vs. DMSO (0.5%); * *p* ≤ 0.05 vs. ACh (10 μM).

Interestingly, the effect of *C. palustre* extracts on the ACh-induced colonic smooth muscle activity was opposite to that on the spontaneous activity; CP1 and CP2 exerted a spasmolytic effect (Figure 5). The same applied for the ether fraction (CP4), which also caused a spasmolytic effect. In addition, the CP5 and CP6 fractions produced a dose-dependent myocontractile effect on colon specimens exposed to acetylcholine (Figure 5). The evoked effect was not clear in character or magnitude only for CP3, ranging around those of the control reaction produced by ACh (Figure 5). The range of doses in which CP1 produced a spasmolytic effect was the widest among all the extracts and ranged from 0.0005 to 0.1 mg/mL. The response was dose-dependent and ranged from 88.32 ± 5.88 to 60.39 ± 4.04% of the reaction to ACh exposed to DMSO (0.5%) for 0.0005 and 0.1, respectively. CP2 clearly weakened the ACh-produced contraction of the colonic smooth muscle in a dose range of 0.01–0.1 mg/mL, and the response reached 74.92 ± 14.59% to 58.93 ± 7.05%. CP4 produced a spasmolytic effect on colon specimens exposed to acetylcholine in a dose range of 0.001–0.1 mg/mL. The reaction ranged from 86.45 ± 6.85% to 72.03 ± 6.97% of the smooth muscle reaction to ACh for CP4 used at concentrations of 0.001 and 0.1 mg/mL, respectively. The administration of CP5 caused a myocontractile effect in a dose-dependent manner in the range of 0.0001–0.1 mg/mL. The response ranged from 126.06 ± 7.80 to 154.55 ± 13.53% of the reaction to ACh for the lowest and the highest concentrations, respectively. CP6 clearly enhanced the ACh-produced contraction of the colonic smooth muscle in a dose-dependent manner in a wide dose range (0.00005–0.1 mg/mL). The reaction ranged from 121.32 ± 9.37% to 155.80 ± 7.15% of the smooth muscle reaction to ACh for CP6 used at concentrations of 0.00005 and 0.1 mg/mL, respectively.

## 3. Discussion

Although the pathogenesis of DGBIs is not fully understood, they are certainly associated with motor disorders [1]. Clinical signs include symptoms related to the reduction or increase in gastrointestinal motility; therefore, gastrointestinal motility modifiers are used in FGID pharmacotherapy [30,31]. Considering the morphological and functional similarities between the human and porcine gastrointestinal tracts [32,33], a pig experimental model was chosen to conduct these studies. Previous research has shown that in vivo experiments can be successfully replaced by ex vivo techniques that rely on isolated intestinal strips [34]. Intestinal fragments can exhibit spontaneous and induced contractility when maintained under conditions that mimic in vivo conditions [35]. When designing our experiment, the current standards of society and the prevailing tendency for researchers to use alternative models rather than live animals were also considered.

In today’s society, plant-derived products, such as cosmetics, medicines, and supplements, are gaining in popularity. An increasing number of patients use alternative medicines in therapy, including those suffering from DGBIs. As indicated in the literature, herbal preparations are the most frequently used alternative treatment methods by patients with IBS [36]. Herbal medicines are also effectively used in relieving the symptoms of functional dyspepsia, functional diarrhoea, or functional constipation [37]. Bearing in mind the growing popularity of herbal medicines and supplements, we decided to investigate the usefulness of *C. palustre* in an ex vivo gut contractility model. Using an LC-DAD-MS analysis, it was possible to characterise the phytochemical profile of CP1–CP3 extracts and CP4–CP6 fractions obtained from *C. palustre*. As a result of the analysis, 15 polyphenols were found, including four dominating flavonoid compounds, such as apigenin (API), luteolin (LUT), apigenin 7-*O*-glucuronide (A7GLC), and chrysoeriol (CHRY).

The results indicate that *C. palustre* extracts and fractions together with their flavonoid constituents are potent modifiers of colon contractility. Modifying effects were observed on spontaneous and ACh-induced activity. The use of different extraction methods enabled us to identify various effects which were dependent on the composition of each preparation. A detailed comparison of the effects induced by the extracts (CP1–CP3) revealed that the highest potency of contractile activity is attributed to CP3. All our three extracts (CP1–CP3) are rich in API and A7GLC, which supports the hypothesis that these phytoconstituents are responsible for or contribute significantly to the total effect of the extracts. On the other hand, the presence of CHRY, which was the most myorelaxant flavonoid, was confirmed mainly in CP1. Most likely, the relaxant character of CHRY and LUT was covered by the more significant prokinetic effects of API and A7GLC. In contrast, the dominant role of API and A7GLC on CP1–CP3 activity is contradicted by the results obtained from trials with pharmacologically induced contractility. As mentioned earlier, all tested extracts (CP1–CP3) showed a spasmolytic effect in the case of ACh-induced activity. CP1 and CP2 are rich in CHRY, which exhibited the strongest spasmolytic effect of all the tested flavonoids. In contrast, CP3 contained significantly less CHRY than the other two extracts. This may suggest the dominant role of CHRY in producing the spasmolytic effect of extracts in the case of ACh-induced contractility. The LC-DAD-MS analysis also showed that all the tested extracts and fractions, except for CP4, also contained a significant amount of chlorogenic acid. At the same time, the research conducted by our team demonstrated the spasmodic effect (both spontaneous and ACh-induced) of chlorogenic acid on swine colon specimens consisting of longitudinal smooth muscle. The obtained results may also indicate the role of chlorogenic acid in the prokinetic activity of CP1–CP3, CP5, and CP6 towards spontaneous colon activity. The absence of chlorogenic acid in CP4 may also explain the opposite, i.e., the myorelaxant effect of this fraction towards ACh-induced contractility. In the case of the fractions (CP4–CP6), there was no clear difference in the force of the myocontractile effect produced on the spontaneous and induced motor activity of the colon because their potency was similar. The only significant difference was that CP4 caused a myorelaxant effect in the case of ACh-induced contractility, although it increased the spontaneous contractility of the colon. The comparison of the flavonoid content in specific fractions revealed that A7GLC was not detected in CP4. Bearing in mind its potent myocontractile effect on spontaneous and induced motoric activity, the absence of A7GLC (and chlorogenic acid) may at least partially explain the myorelaxant character of CP4. However, the absence of chlorogenic acid in CP4 may refute the hypothesis regarding its inhibition of the contractility of porcine colonic smooth muscle. The hypothesis of the dominant role of A7GLC is confirmed by the results obtained for CP5 and CP6, which, similar to this flavonoid, markedly enhanced the magnitude of spontaneous and ACh-induced contractility.

The single flavonoids API and A7GLC exhibited a stimulatory effect on spontaneous and induced motor activity, while LUT and CHRY weakened the colonic smooth muscle contractility (both spontaneous and ACh-induced). When comparing API and A7GLC, the former seems to be the ingredient with greater potential, as it functions more strongly and in a wider range of doses. CHRY, on the other hand, was more potent and over a wider dose range than LUT. The mechanism behind the spasmolytic effect of LUT on the smooth muscle of the colon has been thoroughly described. It is based on the inhibition of L-type calcium channels [38]. Several studies conducted on guinea pig ileum have also confirmed the antispasmodic effect of luteolin [39,40]. The antispasmodic effect of LUT was confirmed by Sandraei et al. [41] who showed the impact of the flavonoid on GI smooth muscle by the inhibition of protein kinase C activity based on the reduction in Ach- and KCl-induced contractions [41]. In addition, luteolin is a nonselective competitive inhibitor of phosphodiesterases which could also play a role in its antispasmodic activity [42]. However, studies conducted on porcine jejunum and bovine stomach have demonstrated the spasmodic effects of luteolin [34,43]. In studies performed on the jejunum of rabbits and rats, the spasmolytic effect of CHRY has been demonstrated. CHRY was found to exhibit its antispasmodic effects through K^+^ channel activation [39] and by blocking calcium influx through voltage-dependent calcium channels [44]. Contrary to our results, other authors describe the effect of API as spasmolytic [40,45,46]. Generated data reveal the involvement of L-type voltage-dependent Ca2+ channels in apigenin-induced gastric relaxation [47]. However, these studies were performed in the guinea pig jejunum [40,48] and in a mouse model [45,47]. Our study presents the initial documentation of how A7GLC affects gastrointestinal contractility.

Furthermore, prior research on flavonoids has suggested that, apart from their impact on peristalsis, these compounds possess other properties that render the plants containing them as promising candidates for the treatment of DGBIs. It has been demonstrated that flavonoids can effectively suppress the production of inflammatory mediators. Luteolin is proven to inhibit the production of interleukins (IL-1β, IL-2, IL-6, IL-8, IL-12, and IL-17), tumour necrosis factor α (TNF-*α*), interferon (IFN-*β*), and granulocyte-macrophage colony-stimulating factor, along with some chemokines, including eicosanoids (prostaglandin and leukotriene) [49]. Luteolin, one of the polyphenolic compounds extracted from *Perilla frutescens*, was found to inhibit the production of TNF-α and interleukins (IL-1, IL-6, and IL-17A,) while apigenin reduced IL-17A secretion and boosted the anti-inflammatory cytokine IL-10 [50]. Research conducted on a luteolin-rich extract of *Serpylli herba* has revealed its ability to inhibit the release of *β*-Hexosaminidase and consequently its ability to modulate mast cell degradation [10]. In their study, Docsa and co-workers conducted a comprehensive review of the influence of inflammatory mediators on the emergence of gastrointestinal motility disorders. They reached the conclusion that, even though the precise mechanisms through which inflammation impacts peristalsis remain not fully elucidated, cytokines indeed exert an influence on gastrointestinal motility, with the potential to either accelerate or decelerate it [51]. With this fact in mind, it is reasonable to consider that extracts from *C. palustre*, known for their abundance of flavonoids that inhibit the activity of inflammatory mediators, could emerge as a promising candidate for managing peristalsis disorders associated with this underlying condition. Moreover, it has been demonstrated that supplementation with flavonoids, such as luteolin and apigenin, assists in reshaping and enriching the gut microbiota, a significant factor in preserving the optimal functioning of the gut and gut–brain axis [52,53,54]. It is proven that gut microbiota play a significant role in modulating GI motility [55,56]; therefore, apigenin- and luteolin-rich extracts from *C. palustre* show promise as options for addressing DGBIs.

## 4. Materials and Methods

### 4.1. Chemical Solvents, Reagents, and Standards

The reference substances used in the experiments were acetylcholine chloride (ACh), isoproterenol (Isop), and dimethyl sulfoxide (DMSO) (Sigma–Aldrich, St. Louis, MO, USA), which were utilized as the vehicle for water-insoluble preparations. CaCl_2_ (Merck, Darmstadt, Germany), NaH_2_PO_4_ (Fluka Chemie, AG, Buchs, Switzerland), NaCl, KCl, MgSO_4_, NaHCO_3_, and glucose (Avantor Performance Materials, Gliwice, Poland) were used to prepare the incubation media. API, A7GLC, LUT, CHRY, and all extracts and fractions of *C. palustre* except for the aqueous extract were dissolved in 0.5% DMSO. The aqueous extract (CP3) was dissolved in the incubation medium. Modified Krebs–Henseleit solution (M K–HS) containing NaCl (123.76 mM), NaHCO_3_ (14.5 mM), glucose (12.5 mM), KCl (5 mM), KH_2_PO_4_ (2.75 mM), CaCl_2_ (2.5 mM), and MgSO_4_ (1.156 mM) was freshly prepared on the day of the experiment and used as a transportation and incubation medium. pH stability within 7.35–7.45 was ensured by maintaining a constant temperature of 37 °C and continuous bubbling with carbogen (95% O_2_ and 5% CO_2_). A POLWATER DL3-100 unit (Labopol, Kraków, Poland) was used to obtain ultrapure water (UPW). Acetonitrile Optima (LC/MS grade) was purchased from Fisher Scientific (Loughborough, UK). Formic acid (FA) was purchased from Avantor (Gliwice, Poland). Chrysoeriol (3′-*O*-methyl-luteolin) (CHRY) was purchased from Sigma–Aldrich (St. Louis, MO, USA). The standards 4-*O*-caffeoylquinic acid and 3,5-*O*-dicaffeoylquinic acid used for the LC–MS analysis were purchased from BIOKOM (Janki, Poland). Apigenin (API), apigenin 7-*O*-glucuronide (A7GLC) and luteolin (LUT), isokaempferide 7-*O*-glucoside, apigenin 7-*O*-(6”-*O*-methyl)-glucuronide, apigenin 7-*O*-glucuronide, luteolin 7-*O*-glucoside, kaempferol, eriodictyol 7-*O*-glucoside, and 5-*O*-caffeoylquinic acid (purity > 96%) were isolated previously. Final purification of A7GLC was carried out using a Waters HPLC system (components 2707, 2998, 1525, 1525µ) and Empower 3 build 3471 software with a Waters WFC III fraction collector (Milford, MA, USA) [28,57,58].

### 4.2. Plant Material

The specimens used in the study were flower heads of *C. palustre* (Podlaskie, Poland; GPS: 53°15′19.4” N 23°27′57.3” E) consisting of dark purple tubular flowers with purple-tipped bracts. The blossoms were collected when beginning to flower and did not have developed seeds at this stage. Plant material identity was evaluated morphologically in comparison to reference data [59]. The Herbarium of the Department of Pharmacognosy at the Medical University of Bialystok, Poland held a voucher specimen (No. CP 06014). The plant material was dried immediately after harvesting, in a shaded, well-ventilated room.

### 4.3. Extraction Procedure for Preparation of Crude CP1-CP3 Extracts and CP4-CP6 Fractions

Initially, purified plant material (120 g) was exhaustively etched with MeOH and 50% MeOH under reflux. The obtained extracts were combined, and the organics were removed under vacuum and lyophilized. The freeze-dried combined extracts were prepared by fractionation by liquid–liquid extraction with Et_2_O (100 × 100 mL), EtOAc (100 × 250 mL), and *n*-BuOH (110 × 250 mL). The combined organic layers were evaporated to dry to yield 0.79 g of Et_2_O (CP4), 1.8 g of EtOAc (CP5), and 4.41 g of *n*-BuOH (CP6) fractions. Ultrasound-assisted extraction (30 g, 40 °C, five times, 30 min) was applied to prepare the following overall extracts: methanol (CP1), 50% methanolic (CP2), and aqueous (CP3). Finally, these preparations were lyophilized to yield 3.92 g of CP1, 5.9 g of CP2, and 6 g of CP3.

### 4.4. Phytochemical Characterization of Extracts and Fractions by LC-PDA-HRMS

The separation of metabolites was guided by conditions previously described with modifications [60]. The mobile phase was as follows: H_2_O (A) and MeCN (B) both with 0.1% HCOOH using the following gradients: 0–1.5 min, 5% B; 20 min, 20% B; 30 min, 22% B; 60 min, 55% B; and 75% B at 70 min of gradient, then 10 min of equilibration. The flow rate was 0.2 mL/min, and the thermostat temperature was 25 °C. The values of absorption for constituents were matched with the values (280, 340, and 360 nm) of the UV-Vis chromatograms. Additionally, by comparing retention times (R_t_) and spectra (UV, MS) with those of reference compounds and published data, the presence of identified constituents in extracts was confirmed.

### 4.5. Tissue Collection and Preparation

Pharmacological analyses were carried out with an alternative study model of swine colon specimens that was presented previously by Mendel et al. [34]. In brief, colon samples collected from healthy adult pigs enabled us to acquire full-thickness strips of 5 × 15 mm, which further were cut out parallel to the longitudinal muscle fibres.

### 4.6. Assessment of Smooth Muscle Activity

**Figure 6:**
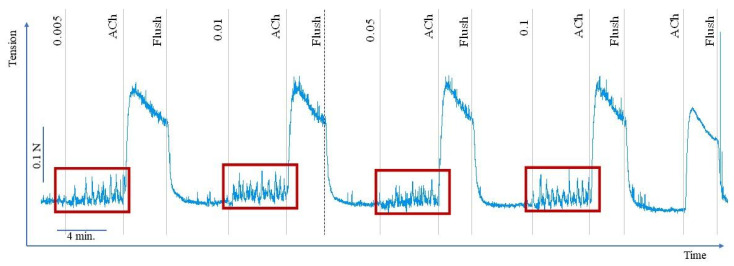
Sample recordings of porcine colon specimens’ response to aqueous extract of *C. palustre* (CP3)—significant increase in the spontaneous contractility. ACh—acetylcholine, Flush—flushed with fresh modified Krebs–Henseleit Solution. The blue line indicates the motoric activity of colon smooth muscle. The elements framed in red indicate remarkable increase of the spontaneous activity induced by *C. palustre* (CP3) application.

After preparation, each muscle preparation was suspended in an individual organ bath chamber (Organ Schuler Bath, Hugo Sachs Elektronik, March, Germany) filled with 5 mL of modified Krebs–Henseleit solution (38.5 °C, continuous bubbling with carbogen 95% O_2_ and 5% CO_2_) to mimic in vivo conditions of intestinal smooth muscle. The strips were attached to metal hooks on one side and to an isometric force transducer (F30, type 372, Hugo Sachs Elektronik, March, Germany) on the other. The experiments were performed under isometric conditions under a load of 0.01 N. An analogue-to-digital registration set (PowerLab, ADInstruments, Bella Vista, NSW, Australia), a bridge amplifier (DBA, type 660, Hugo Sachs Elektronik, March, Germany), and Chart v 7.0 program were utilised to register motor activity records of colon specimens (Figure 6).

### 4.7. Experimental Sequence

**Figure 7:**
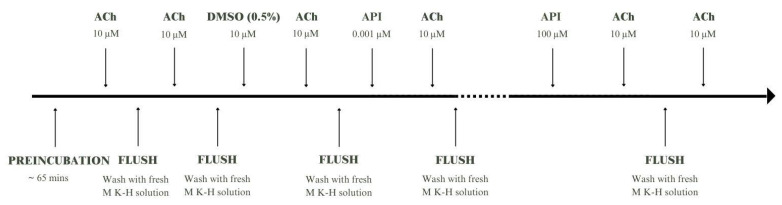
Graphical diagram of the experimental sequence using the example of apigenin (API).

Each experiment started with approximately 65 min of preincubation to stabilise the samples. During the first 45 min of this phase, the tissues were suspended in a no-tension manner. Every 15 min, the chambers were washed with fresh M K–H solution. After that period, a tension of 0.005 N was applied and then increased to 0.01 N after another 15 min. When the spontaneous work of the muscles stabilised, acetylcholine at a concentration of 10 μM was administered to each chamber. Approx. 3 min after each ACh administration, the chambers were washed with fresh M K–H solution. Only the strips that adequately responded to the double administration of the reference contractile substance (ACh) and displayed clear spontaneous contractility were qualified for further experimentation. After spontaneous activity was stabilised, each strip was treated with DMSO (0.5%), and then ACh (10 μM) was added again after 3 min. The reactions to DMSO and ACh + DMSO were then used as a control response to analyse the effects of flavonoids and the extracts/fractions on spontaneous and ACh-induced activity in the colonic smooth muscles, respectively. Once the motility stabilised, flavonoids (API, A7GLC, LUT, and CHRY) and the extracts/fractions (CP1-CP6) were administered in a noncumulative manner in a concentration range of 0.001–100 μM and 0.00001–0.1 mg/mL, respectively. After 5 min of preincubation in the presence of a flavonoid or extract/fraction, ACh (10 μM) was administered. Thorough rinsing with fresh M K–H solution was performed before the next concentration of the tested substance was added. At the end of the experiment, reference substances (ACh, 10 μM and Isop, 1 µM) were applied to validate the reactivity of the preparations (Figure 7). Each flavonoid and extract/fraction of *C. palustre* was tested on a minimum of five colon segments from at least five different animals.

## 5. Conclusions

Our study suggests that selected preparations and flavonoids from *C. palustre* have a pronounced motility-regulating effect. However, the exact mechanism of these interactions remains undiscovered and requires further investigation. To understand the background of the effect of *C. palustre* and its main phytoconstituents on colon contractility, as well as to address the lack of satisfactory methods for treating DGBIs, future trials should aim for the verification of its utility in DGBIs. Bearing in mind its demonstrated antioxidant and antibacterial effects, *C. palustre* and its utility in DGBI patients should be further studied. Further investigations are required to examine the observed effects of these substances on gastrointestinal movements in vivo.

## Figures and Tables

**Table 1:** Principal constituents found by LC-HRMS in extracts/fractions of *C. palustre* flower heads.

No.	Rt (min)	UV Spectra (λ_max_ nm)	Observed ^A^	Δ (ppm) ^B^	Formula	Fragmentation ^C^	Compounds	Presence in Extracts/Fraction					
								CP1	CP2	CP3	CP4	CP5	CP6
1	2.29	**248**, 270, 345	191.01957	−0.65	C_6_H_8_O_7_	191	organic acid derivatives ^F^	x	x	x			x
2	7.23	**260**, 295	153.02000	3,66	C_7_H_6_O_4_	153	phenolic acid derivatives			x	x		
3	8.13	**280**	315.10854	1.22	C_14_H_20_O_8_	203, **315**	phenolic acid derivatives						x
4	10.5	245 *sh*, 295 *sh*, **325**	353.08918	3.85	C_16_H_18_O_9_	**353**, 134	3-*O*-caffeoylquinic acid ^R^			x			x
5	12.39	270	443.19227	0.66	C_21_H_32_O10	215, **443**	phenolic acid derivatives						x
6	14.32	245 *sh*, 295 *sh*, **325**	353.08833	1.5	C_16_H_18_O_9_	**353**, 191	5-*O*-caffeoylquinic acid ^S^	x	x	x	x	x	x
7	15.32	245 *sh*, 295 *sh*, **325**	353.08930	4.14	C_16_H_18_O_9_	**353**, 179	4-*O*-caffeoylquinic acid ^S,R^	x	x	x	x	x	x
8	17.04	290, 312	337.09462	3.33	C_16_H_18_O_8_	**337**, 191	5-*O-p*-coumaroylquinic acid ^R^	x	x			x	
9	17.66	265, 345	623.12809	4.21	C_27_H_28_O_17_	284, 447, **623**	flavone derivative ^F^	x		x			x
10	19.72	**264**, 343	607.13046	3.76	C_27_H_28_O_16_	607	unknown		x	x			x
11	20.57	245 *sh*, 295 *sh*, 327	367.10349	4.62	C_17_H_19_O_9_	**367**, 179, 135	4-*O*-feruloylquinic acid ^R^					x	
12	21.55	255, 282, **344**	463.08820	3.62	C_21_H_20_O_12_	300, **463**	flavone *O*-hex ^F^	x	x			x	x
13	22.74	**283**, 335	449.11088	4.64	C_21_H_22_O_11_	287, **449**	eriodictyol *O*-hex isomer ^F^	x			x	x	x
14	22.82	**283**, 335	449.10894	4.32	C_21_H_22_O_11_	287, **449**	eriodictyol 7-*O*-glucoside ^S^	x	x		x	x	
15	22.63	255, 267 *sh*, **348**	447.09329	4.57	C_21_H_22_O_11_	283, **447**	luteolin 7-*O*-glucoside ^S^	x	x			x	
16	23.67	255, 267 *sh*, **348**	447.09329	4.57	C_21_H_22_O_11_	283, **447**	luteolin *O*-hex isomer ^F^	x	x		x	x	
17	24.39	255, 267 *sh*, **342**	461.07255	6.55	C_21_H_18_O_12_	285, **461**	flavone *O*-uronide derivatives ^F^	x	x	x		x	x
18	26.49	**264**, 347	491.08311	5.73	C_22_H_20_O_13_	315, **447**	cirsimaritin 4’-*O*-glucoside ^F^			x		x	
19	26.58	246, 296, **327**	515.12266	5.05	C_25_H_24_O_12_	191, 353, **515**	3,4-*O*-dicaffeoylquinic acid ^F^	x	x	x			
20	26.67	246, 296, **327**	515.12240	6.03	C_25_H_24_O_12_	191, 353, **515**	3,5-*O*-dicaffeoylquinic acid ^S,F^			x			
21	26.72	246, 296, **327**	515.11950	5.09	C_25_H_24_O_12_	191, 353, **515**	dicaffeoylquinic acid isomer ^F^			x	x	x	
22	26.92	246, 296, **327**	515.12212	5.31	C_25_H_24_O_12_	191, 353, **515**	dicaffeoylquinic acid isomer ^F^	x	x	x	x	x	
23	27.18	266, 336	431.10043	4.79	C_21_H_20_O_10_	268, **431**	flavone *O*-hex isomer ^F^	x	x		x	x	
24	27.68	**250**, 295 *sh*, 327	631.13046	3.25	C_29_H_28_O_16_	191, 353, **631**	quinic acid derivatives						x
25	29.14	266, **336**	445.07763	3.98	C_21_H_18_O_11_	269, **445**	apigenin 7-*O*-glc (A7GLC) ^S^	x	x	x	x	x	x
26	29.79	245 *sh*, 295 *sh*, **325**	515.11950	4.63	C_25_H_24_O_12_	515	dicaffeoylquinic acid isomer ^F^				x	x	
27	30.82	266, **350**	461.10894	3.86	C_22_H_22_O_11_	283, **461**	isokaempferide 7-*O*-glu ^S^	x	x		x		
28	31.12	274, **334**	475.08907	2.34	C_22_H_20_O_12_	283, 299, **475**	isokaempferide 7-*O*-glc ^F^	x	x	x		x	x
29	31.25	264, **340**	431.10021	4.52	C_21_H_20_O_10_	284, **431**	flavone derivatives	x	x		x		
30	33.67	274, **334**	475.08820	1.84	C_22_H_20_O_12_	255, 299, **475**	flavone *O*-hex derivatives ^F^	x	x	x		x	x
31	37.36	268, **325**	593.13006	4.62	C_30_H_26_O_13_	593	unknown		x			x	
32	38.4	268, **336**	459.09329	4.8	C_22_H_20_O_11_	269, **459**	apigenin 7-*O*-(6’’-*O*-methyl)-glc ^S^					x	
33	38.48	268, **345**	285.04046	3.48	C_15_H_10_O_6_	285	luteolin (LUT) ^S^				x		
34	44.24	268, 290 *sh*, **338**	269.04609	2.03	C_15_H_10_O_5_	269	apigenin (API) ^S^	x	x	x	x		
35	44.67	266, 29 *sh*, **358**	285.04178	4.39	C_15_H_10_O_6_	285	Kaempferol ^S^	x	x		x		
36	45.69	**266**, 293 *sh*, 350	299.05697	2.89	C_16_H_12_O_6_	299	chrysoeriol (CHRY) ^S^	x	x	x	x		
37	46.56	295, **308**	785.35848	−1.41	C_38_H_58_O_17_	545, 665, **785**	unknown	x	x		x	x	

^A^—Exact mass of [M-H]- ion; ^B^—mass error; ^C^—fragmentation in negative ion mode; *sh*—peak shoulder; bold—most abundant; glu—glucose; glc—glucuronide; hex—hexoside; ^F^—predicted by UV-Vis and MS spectra; ^S^—reference substance; ^R^—according to Cliffort et al. 2003 [29].

## Data Availability

Data are contained within the article.
